# Simultaneous Determination of Five Alkaloids by HPLC-MS/MS Combined With Micro-SPE in Rat Plasma and Its Application to Pharmacokinetics After Oral Administration of *Lotus Leaf* Extract

**DOI:** 10.3389/fphar.2019.01252

**Published:** 2019-10-22

**Authors:** Shuhan Zou, Yuanyuan Ge, Xuanhao Chen, Jin Li, Xuejing Yang, Hui Wang, Xiumei Gao, Yan-xu Chang

**Affiliations:** ^1^Tianjin State Key Laboratory of Modern Chinese Medicine, Tianjin University of Traditional Chinese Medicine, Tianjin, China; ^2^Tianjin Key Laboratory of Phytochemistry and Pharmaceutical Analysis, Tianjin University of Traditional Chinese Medicine, Tianjin, China; ^3^School of Pharmacy, Harbin University of Commerce, Harbin, China

**Keywords:** *lotus leaf*, micro-solid phase extraction, pharmacokinetics, HPLC-MS/MS, PEP-2

## Abstract

An environment-friendly and efficient method for simultaneous determination of five alkaloids (nunciferine, O-nornuciferin, liriodenine, armepavine, and pronuciferine) in rat plasma was established by HPLC-MS/MS associated with micro-solid phase extraction (micro-SPE). The plasma sample was pretreated by using micro-SPE columns filled with polymer materials PEP-2 and eluted by little organic solvent (400 µl acetonitrile). The five alkaloids were separated with acetonitrile and 0.1% formic acid aqueous solution on Eclipse plus C_18_ column. The mode of positive electrospray ionization was used to measure the analytes in multiple-reaction monitoring (MRM). The determination coefficients (R^2^) of the five alkaloids were greater than 0.99. The lower limit of quantification (LLOQ) of O-nornuciferin, liriodenine, and armepavine was 0.5 ng·ml^−1^, and that of nunciferine and pronuciferine was 1 ng·ml^−1^. The validated method was effectively used for the pharmacokinetics of the five orally administrated alkaloids of *lotus leaf* extract in rat plasma.

## Introduction

*Lotus leaf*, dry leaf of the lotus (species *Nelumbo nucifera* Gaertn of genus *Nelumbo* Adans. of family Nelumbonaceae A.Rich.), is one of the traditional Chinese herbal medicines compiled in Chinese Pharmacopoeia (Chinese Pharmacopoeia Commission, 2015). *Lotus leaf* is used not only as traditional Chinese medicine to resolve summer heat, clear heat, and stanch blood but also as tea to reduce lipid and lose weight in China (Mukherjee et al., 2009; Ho et al., 2010). Alkaloids and flavonoids are the main chemical constituents in *lotus leaf* (Paudel and Panth, 2015; Sharma et al., 2016). The previous studies have indicated that alkaloids in *lotus leaf* possess various biological activities including lipid lowering (Fan et al., 2013; Ye et al., 2014; Ye et al., 2016) and anti-inflammatory (Wang et al., 2015). Recently, phytochemical studies demonstrated that nuciferine and pronuciferine could alleviate dyslipidemia and liver steatosis and inhibit lipogenesis in 3T3-L1 adipocytes (Guo et al., 2013; Ma et al., 2015). Liriodenine, O-nornuciferin, and armepavine exhibit the activities of antioxidant and antimicrobia antifibrotic (Costa et al., 2010; Weng et al., 2012). In addition, there were only a few reports for pharmacokinetic studies of one or two alkaloid compounds after oral administration of *lotus leaf* extract (Gu et al., 2014; Xu et al., 2015; Geng et al., 2018; Ye et al., 2018). However, the pharmacokinetic property of one or two alkaloids does not represent the metabolic regulation of the whole *lotus leaf* *in vivo*. Therefore, it is necessary to perform the pharmacokinetic studies of multiple components for clarifying the work mechanism of *lotus leaf*.

The sample preparation is an important step in the process of pharmacokinetic study because the responses of analytes are influenced by sample preparation method. Biological samples usually were pretreated by liquid–liquid extraction and solid phase extraction (SPE) in pharmacokinetic study (Adamowicz et al., 2013; Shanks et al., 2015; Schaefer et al., 2015; Ozturk et al., 2015). In recent years, more and more novel methods of sample preparation were used, especially micro-SPE (Wang et al., 2016; Margalef et al., 2014; Montesano et al., 2017; Iqbal et al., 2018). Compared with these methods, micro-SPE has advantages of less sorbent, simple operation, and more miniaturization. Moreover, the choice of sorbent played an important role in scope and sensitivity of analyzed compounds. PEP-2 is a polymer material that could be infiltrated by water and resistant to pH 0–14. It can avoid the situation of low recovery and unstable experimental result caused by the drying of the traditional silica C_18_ material in elution. Therefore, application of PEP-2 is especially beneficial for repeatability and sensitivity of experiment in biological sample preparation.

**Figure 1:**
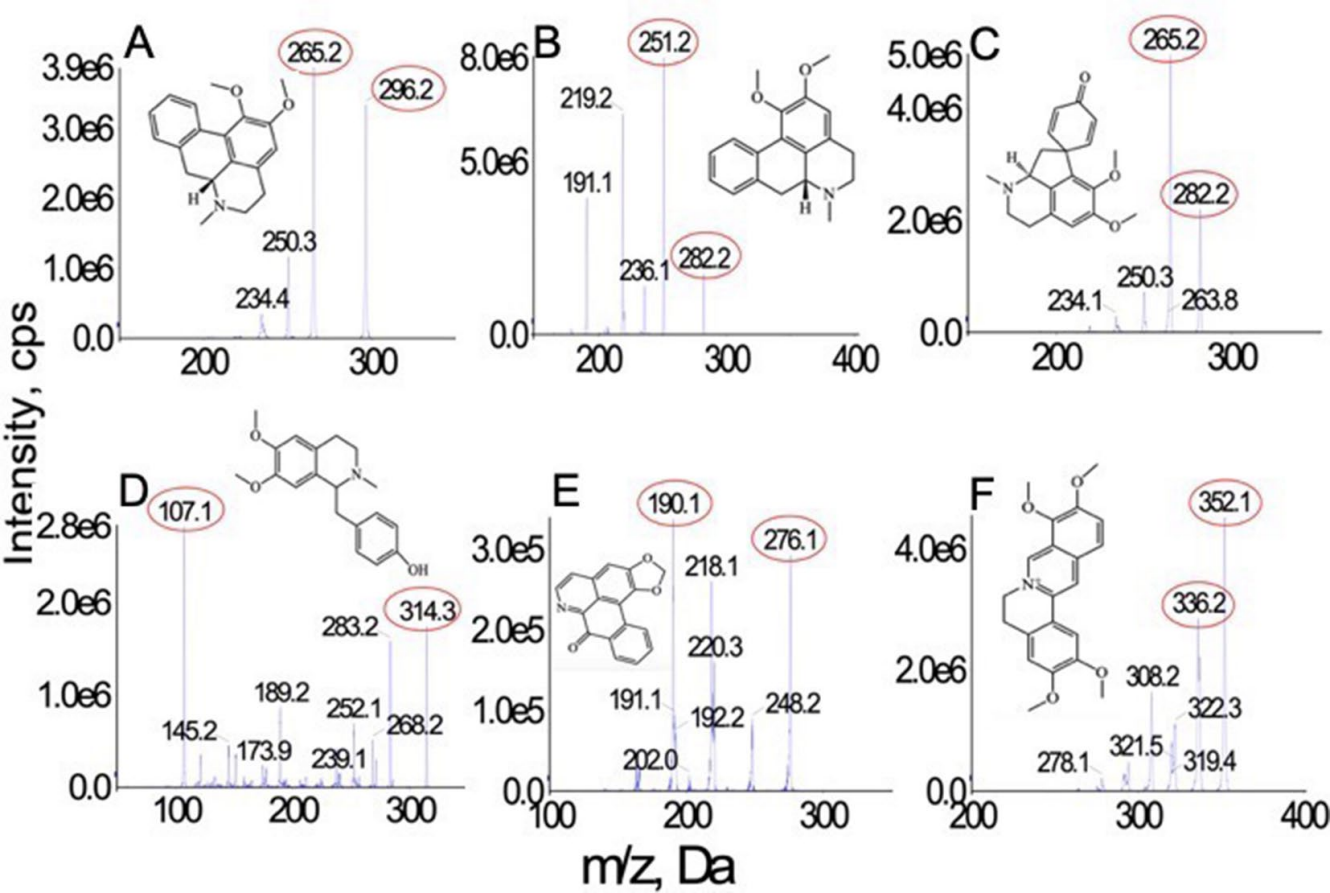
Chemical structures and MS spectra of nunciferine **(A)**, O-nornuciferin **(B)**, pronuciferine **(C)**, armepavine **(D)**, liriodenine **(E)**, and palmatine chloride **(F)**.

In this study, an efficient method was established by combining HPLC-MS/MS with micro-SPE for simultaneous determination of five alkaloids (nunciferine, O-nornuciferin, liriodenine, armepavine, and pronuciferine) in rat plasma in the pharmacokinetic study of *lotus leaf* (**Figure 1**). Moreover, three alkaloids including O-nornuciferin, liriodenine, and pronuciferine in rat plasma were firstly determined in the pharmacokinetic study of *lotus leaf*. The novel method was effectively used to provide the pharmacokinetic outlines for clinical study of *lotus leaf*.

## Experimental

### Materials and Reagent

The samples were purchased from a Tongrentang Chinese Medicine store in Tianjin. The voucher number for the plant (GZTM 0009013) was provided by Chinese Virtual Herbarium.

Nunciferine, O-nornuciferin, liriodenine, armepavine, pronuciferine, and internal standard (IS) palmatine chloride were obtained from Chengdu DeSiTe Biological Technology Co., Ltd. Their purities were more than 98%. Acetonitrile and methanol were achieved from Fisher (USA). The formic acid was obtained from Tedia. The deionized water was provided by the Mili-Q system (Millipore Corp., USA). Six kinds of sorbents including ODS C_18_–N, C_8_, cation exchange resin PCX, polymer materials PEP-2, macroprorous resin HP-20, and strong cation exchange resin SCX were purchased from Agela Tec. Co. Ltd (Tianjin, China). Empty cartridge (1 ml) and PE frit (10 μm) were obtained from Agela Tec. Co. Ltd (Tianjin, China).

### IS Selection

Five alkaloids were simultaneously determined. Palmatine chloride was selected for IS according to the similar structure and no interference from endogenous substances. The stock solutions of IS (1 mg·ml^−1^) were obtained by dissolving in methanol.

### Conditions of HPLC-MS/MS

All analyses were performed on Agilent 1200 HPLC system (Agilent Corporation, USA) and API 3200 triple-quadrupole mass spectrometer (Concord, Ontario, Canada) in multiple-reaction monitoring (MRM) with positive ionization mode. Acetonitrile (A) and formic acid aqueous solution (B) (0.1%, v/v) is used to separate the analytes on Eclipse plus C_18_ column (4.6 mm × 100 mm, 1.8 µm) with a guard C_18_ column (2.1 mm × 12.5 mm, 5 µm). The gradient of elution is as follows: 5%–60% A at 0 to 4 min, 60%–70% A at 4 to 5 min, 70%–93% A at 5 to 7 min, 93%–94% A at 7 to 11 min, 94%–5% A at 11 to 12 min, and 5%–5% A at 12 to 17 min. The flow rate and injection volume are 0.3 ml·min^-1^ and 5 µl, respectively. The main parameters of ion source including the ion spray voltage, the curtain gas, and temperature are set at 5000 V, 35 psi, and 450°C, respectively. All MRM parameters are listed in **Table 1**.

**Table 1:** MRM parameters of the five compounds and IS.

Compounds	Q_1_	Q_3_	DP (V)	EP (V)	CE (V)	CXP (V)
Nunciferine	296.2	265.2	33	6.0	20	23
O-nornuciferin	282.2	251.2	42	7.0	20	21
Liriodenine	276.1	190.1	70	3.0	55	15
Armepavine	314.3	107.1	50	3.0	35	9.0
Pronuciferine	282.2	265.2	35	5.0	15	23
Palmatine chloride (IS)	352.1	336.2	56	3.0	35	28

MRM, multiple-reaction monitoring; IS, internal standard; DP, declustering potential; EP, entrance potential; CE, collision energy; CXP, collision cell exit potential.

### Preparation of *Lotus Leaf* Extract

The crude *lotus leaf* (1 kg) was extracted in 95% ethanol (1:8, w/v) for 1 h under the heat reflux method, and then the gruffs was extracted in 60% ethanol (1:8, w/v) for 1 h again. Under a reduced pressure condition, the two filtrates were mixed and concentrated in a rotary evaporator at 60°C, until it was dried with a vacuum dryer at 50°C. The extraction yield was 22.52%.

### Preparation of Standard and Quality Control Solutions

Five reference standards were individually dissolved in methanol to obtain the stock solution at a concentration of 1 mg·ml^−1^, respectively. Certain amounts of standards were mixed up to a solution containing 10 μg·ml^−1^ nunciferine, 10 μg·ml^−1^ O-nornuciferin, 10 μg·ml^−1^ liriodenine, 5 μg·ml^−1^ armepavine, and 5 μg·ml^−1^ pronuciferine with methanol. Then a suit of reference stock solution was diluted with methanol and further diluted with methanol to gain 10 different appropriate concentrations for calibration curve.

Quality control (QC) samples of all analytes were prepared at the lower limit of quantification (LLOQ), low, medium, and high concentration levels (1, 2, 100, and 500 ng·ml^−1^ for nunciferine; 0.5, 1, 40, and 200 ng·ml^−1^ for O-nornuciferin; 0.5, 1, 40, and 200 ng·ml^−1^ for liriodenine; 0.5, 1, 20, and 100 ng·ml^−1^ for armepavine; 1, 2, 100, and 500 ng·ml^−1^ for pronuciferine) by dissolving appropriate mixed standard solutions in methanol. All the related solutions were kept at 4°C.

### Optimization of Micro-SPE Conditions

Six types of sorbents (ODS C_18_-N, C_8_, cation exchange resin PCX, polymer materials PEP-2, macroprorous resin HP-20, and strong cation exchange resin SCX) were optimized as an optimal sorbent in micro-SPE procedure. When this factor was optimized, the other conditions remained the same. The amount of sorbent (5, 10, 15, 20, and 25 mg) were examined for an optimal amount. Then, five concentration levels of methanol regarded as wash solution (5%, 10%, 20%, 30%, and 40%) were measured. Furthermore, the type of eluent (methanol and acetonitrile) and volumes of eluent (200, 300, 400, and 500 µl) were considered to be optimized. In the process of optimizing micro-SPE condition, the blank plasma (100 µl), mixed standard solution (10 µl), and IS solution (10 µl) were vortex-mixed for 1 min and added into the SPE column. The absolute recovery and matrix effect of standard were regarded as the evaluation index.

### Sample Preparation

**Figure 2:**
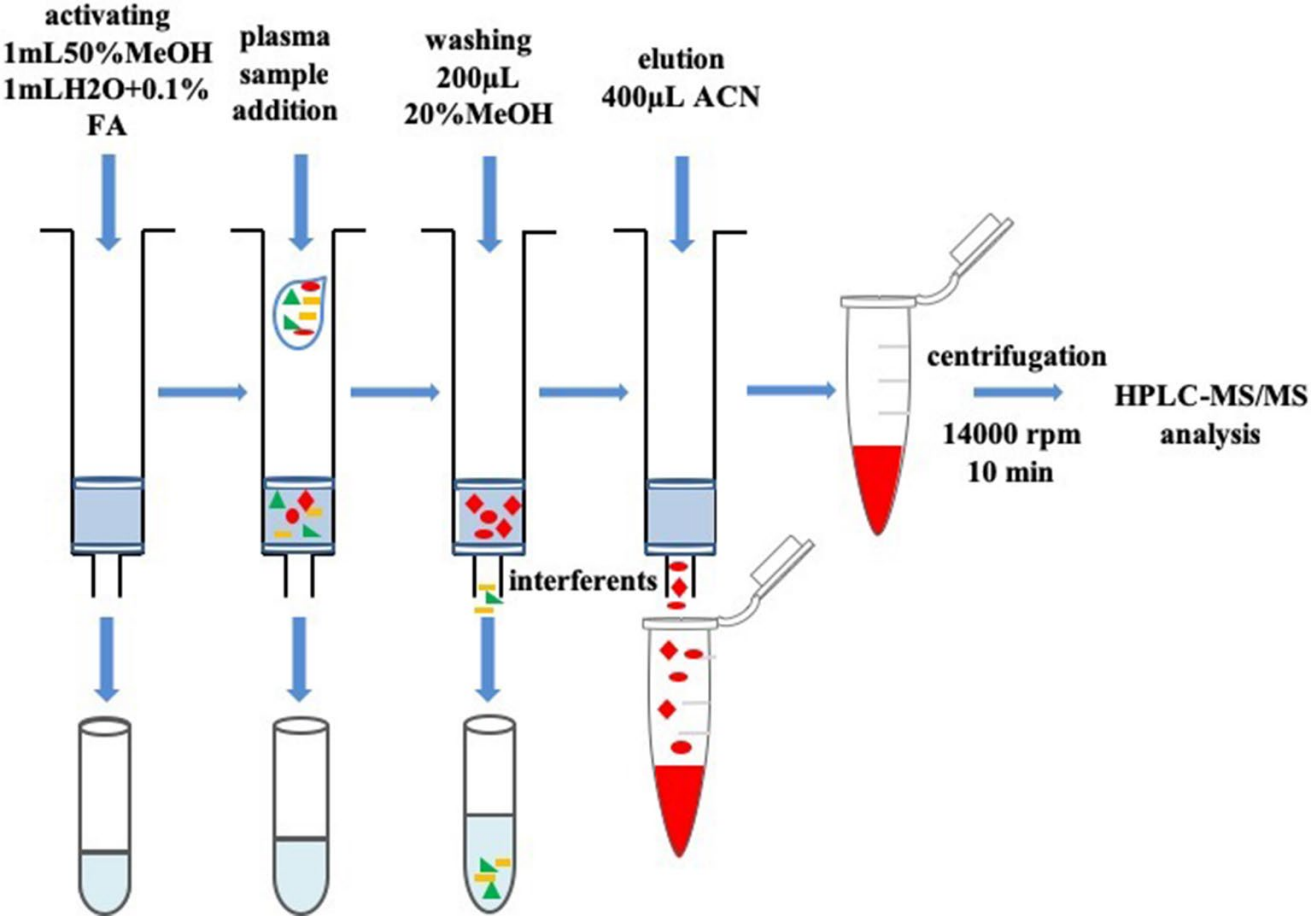
Schema of the plasma preparation and SPE procedure.

The real plasma sample (100 µl) and IS solution (10 µl) were vortex-mixed for 1 min. The sample was carried out with the SPE column (Cleanert PEP-2, 40–60 µm, 60 Å, 10 mg/1 ml; Phenomenex, Torrance, CA, USA). First, SPE columns were activated by 50% methanol in water (1 ml) and 0.1% formic acid in water (1 ml) and were used to activate the SPE column in sequence. Second, columns were washed with 200 µl methanol (20%, v/v) and eluted with acetonitrile (400 µl) after the plasma samples were added. Finally, the eluent was centrifuged for 10 min with 14,000 rpm. Then final solutions were injected into the HPLC-MS/MS and analyzed. The schema of the procedure is shown in **Figure 2**.

### Method Validation

The reproducibility and reliability of the established method were calculated by method validation following the US Food and Drug Administration guidelines [14]. The parameters included precision, accuracy, recovery, carryover effect, and so on.

#### Linearity and Sensitivity

Calibration standard solutions (10 µl) were added into blank plasma with the final concentration ranging from 1 to 1,000 ng·ml^−1^ (1, 2, 4, 10, 20, 50, 100, 250, 500, and 1000 ng·ml^−1^) for nunciferine and pronuciferine and 0.5 to 500 ng·ml^−1^ (0.5, 1, 2, 5, 10, 25, 50, 125, 250, and 500 ng·ml^−1^) for O-nornuciferin, liriodenine, and armepavine, then the mixed samples were processed by micro-SPE. The linearity was evaluated with the peak area ratios of five alkaloids to IS against concentrations using 1/X^2^ weighting. The lowest concentrations were accurately determined as LLOQ in the calibration curves.

#### Specificity

The specificity was evaluated by analyzing blank plasma sample, blank plasma added into five alkaloids at level of LLOQ, and real plasma orally administrated *lotus leaf* extract. All samples were pretreated by micro-SPE and measured.

#### Precision and Accuracy

The QC samples from six different batches at the level of four concentrations (LLOQ, low, medium, and high) were used to assess precision and accuracy within a day and among three different days. The relative standard deviation (RSD) and the percent ratios of the calculated concentration to nominal concentration were used as evaluation index.

#### Stability

The stabilities (including auto-sampler, freeze–thaw cycles, and short-time and long-term stability) of all analytes were obtained by testing QC samples of four concentrations. Moreover, the stabilities for stock and working solution were assessed with mixed standard substance (100 ng·ml^−1^) and the QC sample solutions of four concentration levels, respectively. These solutions were achieved by diluting stock solutions kept at 4°C for 2 weeks. For IS, the evaluated procedures of two stabilities were same as the five alkaloids.

#### Recoveries and Matrix Effects

The recoveries and matrix effects of the five alkaloids were measured by six QC samples at four concentration levels. The recoveries were obtained with dividing the peak areas of the five alkaloids spiked into plasma before micro-SPE elution (A) by those of the five alkaloids spiked into processed blank samples after the micro-SPE elution (B). The matrix effects were achieved with dividing those of the five alkaloids spiked into processed blank samples after the micro-SPE elution (B) by the peak of the five alkaloids in solvent (C). For IS, the evaluated procedure of recovery and matrix effect were same as the five alkaloids.

#### Carryover

The carryover effects were measured by analyzing the plasma samples at LLOQ and the high concentration levels and blank plasma samples successively in three cycles. There was no effect on the experiment from the carryover, when the responses of analytes were less than 20% of the LLOQ response and those of IS were less than 5% of IS working solution response in the blank plasma samples.

### Pharmacokinetics

The pharmacokinetic study was conducted in accordance with the Guidelines for the Care and Use of Laboratory Animals by the US National Institutes of Health and approved by the Animal Ethics Committee of Tianjin University of Traditional Chinese Medicine (permit number: TCM-LAEC2019022, Tianjin, China),. Male Sprague-Dawley rats (240–260 g) were fed in the animal laboratory of the Tianjin University of Traditional Chinese Medicine under the standard conditions. These rats were allowed free to get standard laboratory food and water and conformed for 1 week until 12 h before experiment. These rats (*n* = 10) were fasted, although they were still given water for 12 h, before the oral administration of *lotus leaf* extract. Blood samples (about 250 µl) were achieved in heparinized 1.5-ml polythene tubes at 0, 0.083, 0.25, 0.5, 1, 2, 3, 4, 5, 6, 7, 8, 10, 12, 14, and 24 h after oral administration of 2.4 g·kg^−1^ *lotus leaf* extract. The plasma samples were centrifuged in 10 min at 7000 rpm and kept at −20°C until analyzed.

### Analysis of Data

All of the pharmacokinetic parameters were calculated by one compartment model with DAS 1.0 software (Medical College of Wannan). The maximum drug concentration (C_max_) and the time to reach C_max_ (T_max_) were acquired from observed concentration–time profile for oral administration in plasma. All data were shown as mean ± SD.

## Result and Discussion

### HPLC-MS/MS Optimization

**Figure 3:**
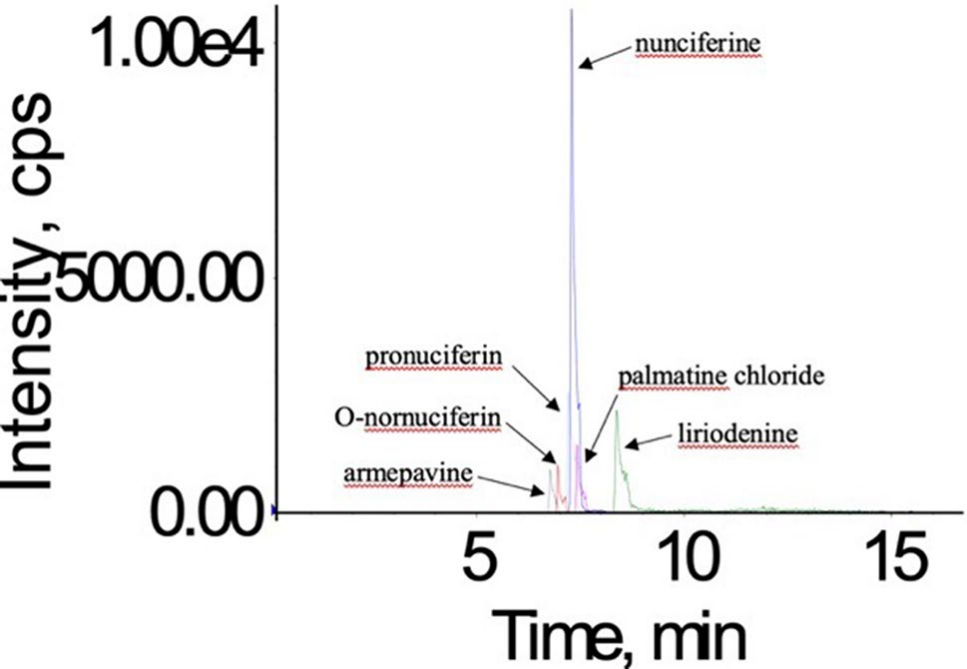
The total ion current chromatogram of nunciferine, O-nornuciferin, pronuciferine, armepavine, liriodenine, and palmatine chloride (IS).

In order to obtain good chromatographic condition, the mobile phase was optimized. Compared to methanol, the mass responses of all analytes were greatly improved by acetonitrile instead of methanol. Moreover, the mobile phase also influenced the ionization efficiency and the response of all analytes. When the 0.1% formic acid was added into the aqueous solution, the five alkaloids possessed a higher response and more symmetrical shape (**Figure 3**).

The standard solutions of all analytes were directly injected into the mass spectrometer with electrospray ionization source. Compared with the sensitivity of analytes under the positive ionization mode, they possessed a higher sensitivity under the negative mode. All analytes were quantified in MRM mode. The precursor and product ion pairs for MRM detection and their corresponding declustering potential, collision energy, entrance potential, and collision cell exit potential values were optimized to obtain the maximum sensitivity and response. All optimized parameters are list in **Table 1**. The product ion san spectra of all analytes are shown in **Figure 1**.

### Quantification of the Five Alkaloids in *Lotus Leaf* Extract

The contents of the five alkaloids in *lotus leaf* extract were measured. The dosages of nunciferine, O-nornuciferin, liriodenine, armepavine, and pronuciferine were 28.8, 9.36, 3.6, 6.12, and 9.41 mg/kg, respectively.

### Optimization of Micro-SPE Conditions

Due to the complicated matrix effect of biological samples, the establishment of an appropriate and efficient preparation method is important to extract and purify analytes. It can enhance the sensitivity of the detection method and reduce the matrix interference. Then, the micro-SPE procedure was affected by some parameters (including the type of sorbent, amount of sorbent, the concentration of wash solution, the type of eluent, and the volume of eluent). All of these parameters were investigated in detail.

#### Optimization of Sorbent Type

**Figure 4:**
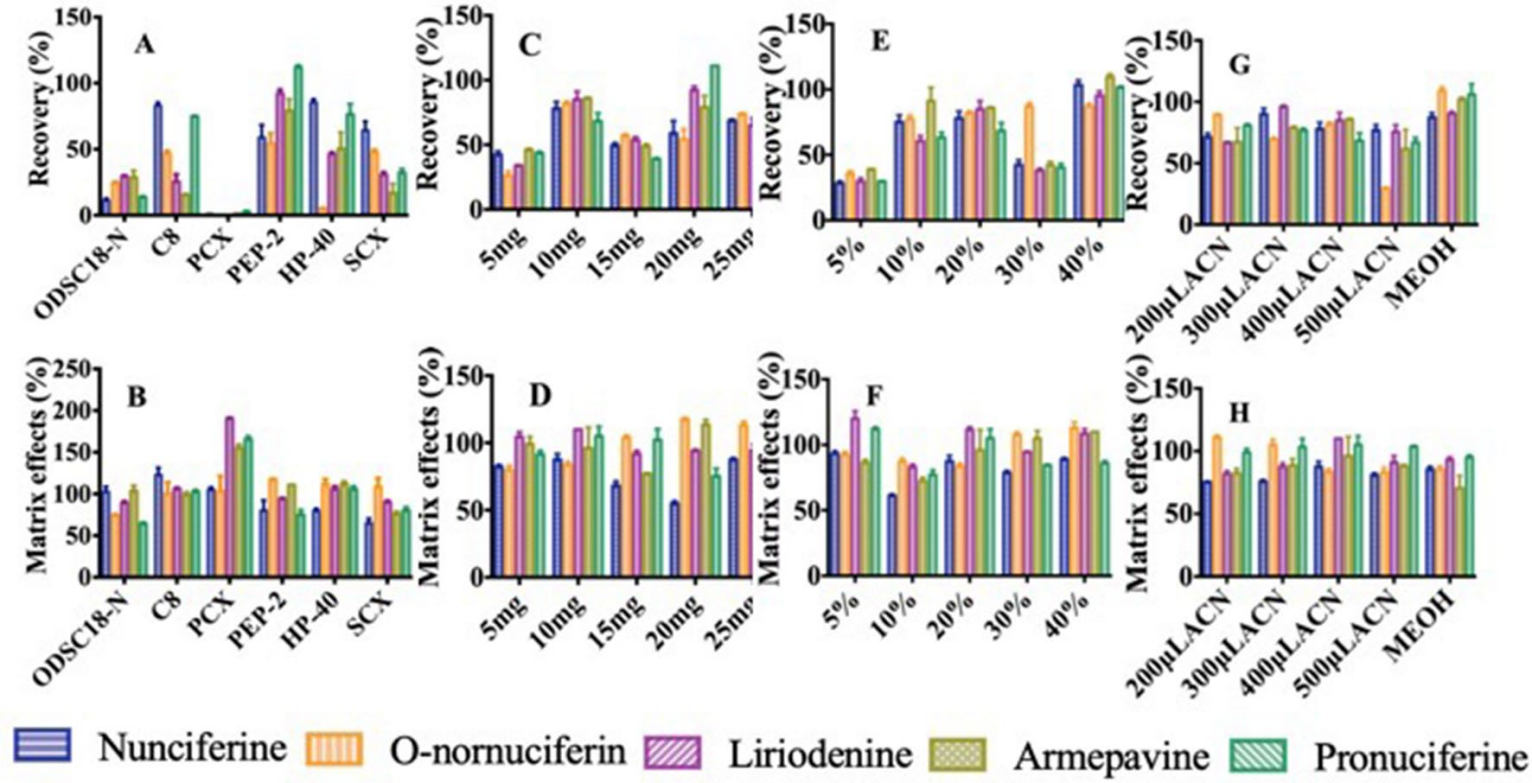
Effects of parameters on recovery and matrix effects of five alkaloids: **(A**, **B)** Type of the sorbent. **(C**, **D)** Amount of the sorbent. **(E**, **F)** Concentration of methanol taken as washing solution. **(G**, **H)** Type and volume of eluent.

The sorbent is one key factor of parameters on extraction ability because the physicochemical properties affect the adsorption capacity for different sorbents. The results indicated that PCX made the recoveries of analytes poorest in the six sorbents, according to the principle of ion exchange (**Figures 4A, B**). When ODSC_18_-N and SCX were taken as sorbent, the recoveries of analytes were in ranges of 10.2% to 32.3% and 11.7% to 69.2%, respectively. Due to the non-polar bonded and hydrophobicity, the recoveries of three alkaloids were in the range of 15.0% to 48.2% when C_8_ was used as sorbent. The recovery of O-nornuciferin was 4.94% when HP-40 was regarded as sorbent. The recoveries and matrix effects of analytes were in ranges of 48.8% to 113% and 71.1% to 118%, respectively, when polymer material PEP-2 was selected as sorbent. That might be related to the bonding of pyrrolidone and carbamido and the wettability in PEP-2 surface. It can avoid the situation of low recovery and unstable experimental result caused by the drying in elution. In addition, the high surface area of polymer suggested that more samples were treated by less sorbent. Therefore, polymer material PEP-2 was used as sorbent in sequential experiment.

#### Optimization of Sorbent Amount

The amount of sorbent influences the contact area of compounds and sorbent, then determines the ability of retaining compounds. The result demonstrated that the recoveries of most analytes increased at first then decreased when the amount of PEP-2 ranged from 5 to 25 mg. Moreover, the recoveries and matrix effects of the five alkaloids were 58.8% to 74.1% and 86.7% to 115%, respectively, when 10 mg of PEP-2 was used (**Figures 4C, D**). That could be related to the adsorption capacity of sorbent reach saturation when the amount of sorbent was 10 mg. Therefore, the optimal sorbent amount was 10 mg.

#### Optimization of Washing Solution

The deproteinization has a great effect on matrix effect influencing the response and selectivity of analytes. The different proportions of methanol (5%, 10%, 20%, 30%, and 40%) were measured for obtaining the optimal proportion of methanol to remove protein. As shown in **Figure 4F**, when 20% methanol was used to remove proteins, the matrix effects of nunciferine, O-nornuciferin, liriodenine, armepavine, and pronuciferine reached 87.6%, 83.6%, 112%, 96.1%, and 105%, respectively. Moreover, when the protein was removed by 20% methanol, the recoveries of these alkaloids reached 78.1%, 81.9%, 85.0%, 86.0%, and 68.4%, respectively (**Figure 4E**). The recoveries and matrix effects of the five alkaloids were satisfactory and sufficient, when the washing solution was 20% methanol. Thus, the optimal proportion of washing solution was 20% methanol.

#### Optimization of Eluent Type and Volume

The type of eluent was key to optimize the extraction ability in pretreatment procedure. As shown in **Figure 4G**, compared with the recoveries of all analytes when methanol was used as eluent, they were in the range of 84.8% to 111% when acetonitrile was taken as eluent. Therefore, acetonitrile was chosen as optimal eluent.

The different volumes of acetonitrile (200 to 500 µl) were measured for obtaining the optimal volume of eluent. Although there was no significance in matrix effects between the four different volumes of eluent (200, 300, 400, and 500 µl), the recoveries of the five alkaloids were statistically significant at different volumes of eluent (**Figures 4G, H**). The results showed that the recoveries of nunciferine, O-nornuciferin, liriodenine, armepavine, and pronuciferine reached 78.1%, 81.9%, 85.0%, 86.0% and 68.4% when the volume of eluent was 400 µl, respectively. Thus, the optimal volume of eluent was 400 µl.

### Method Validation

#### Specificity

**Figure 5:**
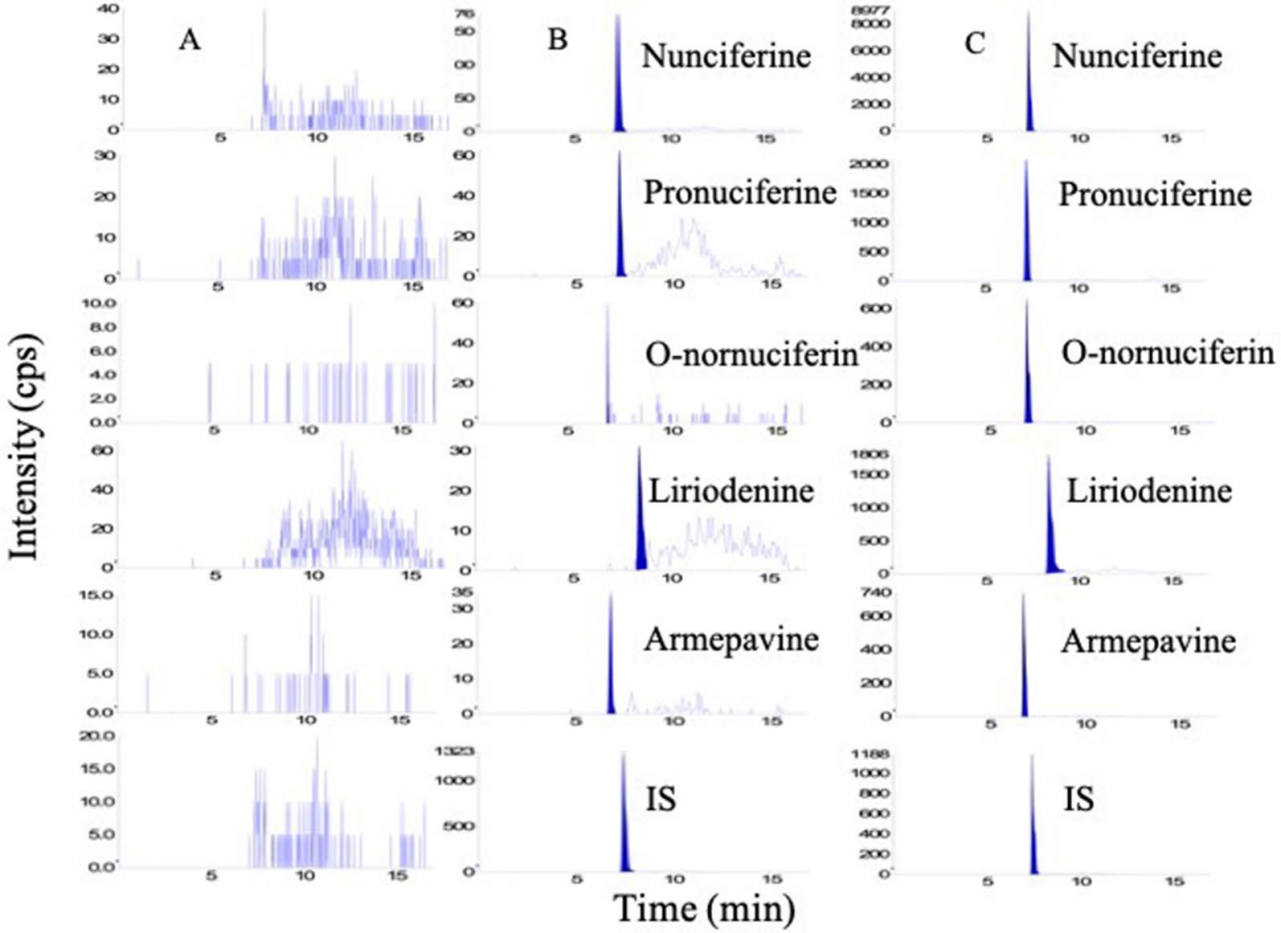
Representative chromatogram of **(A)** blank plasma, **(B)** blank plasma spiked with standard compounds at LLOQs, and **(C)** plasma sample.

The representative chromatograms of analytes are shown in **Figure 5**. There were a good separation and no interference from endogenous substances for all analytes when the retention times of nunciferine, O-nornuciferin, liriodenine, armepavine, pronuciferine, and IS palmatine chloride were 7.29, 6.96, 8.37, 6.77, 7.21, and 7.42 min, respectively.

#### Linearity and Sensitivity

The linearity ranges, LLOQ, correlation coefficients, and equations of calibration curve of the five alkaloids are listed in **Table 2**. The LLOQ of O-nornuciferin, liriodenine, and armepavine was 0.5 ng·ml^−^^1^, and that of nunciferine and pronuciferine was 1 ng·ml^−^^1^. The data indicated that the sensitivity of the established method was sufficiently used for the pharmacokinetics of *lotus leaf* in rats.

**Table 2:** The calibration curves, linearity range, and LLOQ of five compounds (*n* = 6)

Compounds	Regression equation	r	Linearity range	LLOQ (ng/ml)
Nunciferine	Y = 0.0832X + 0.109	0.9938	1–1000	1
O-nornuciferin	Y = 0.0134X + 0.00413	0.9983	0.5–500	0.5
Liriodenine	Y = 0.0602X + 0.0398	0.9971	0.5–500	0.5
Armepavine	Y = 0.0294X + 0.00217	0.9918	0.5–500	0.5
Pronuciferine	Y = 0.0306X + 0.0347	0.9942	1–1000	1

LLOQ, lower limit of quantification.

#### Accuracy and Precision

The values for accuracy and precision of intra-day and inter-day are listed in **Table 3**. The precision was lower than 15%, and the accuracy was in the range of 85.5% to 118%. It was demonstrated that the method was reproducible and precise.

**Table 3:** Intra-day, inter-day, accuracy, and precision, recovery, matrix effect of five compounds (*n* = 6).

Compounds	Concentration (ng/ml-)	Intra-day		Inter-day					
		Accuracy (%)	RSD (%)	Accuracy (%)	RSD (%)	Recovery (%)	RSD (%)	Matrix effect (%)	RSD (%)
Nunciferine	LLOQ	92.4	13.1	93.9	13.25	98.9	7.03	112	6.38
	2	85.7	14.2	94.1	7.36	105	8.95	88.4	7.82
	100	95.5	6.37	99.4	8.11	86.1	3.93	87.0	4.12
	500	85.8	3.56	87.0	1.93	88.6	8.24	90.2	8.48
O-nornuciferin	LLOQ	108	11.9	98.3	8.86	98.0	4.59	100	6.37
	1	111	14.3	102	8.36	102	10.0	94.0	10.9
	40	118	9.12	106	11.9	86.6	3.85	105	6.42
	200	98.0	8.12	96.9	1.83	94.1	5.43	103	5.13
Liriodenine	LLOQ	92.9	8.29	93.6	8.77	103	8.81	100	7.34
	1	90.6	9.52	87.2	3.01	98.7	5.62	109	9.43
	40	109	6.18	102	8.63	86.5	6.22	92.3	9.11
	200	91.2	6.78	93.4	6.03	85.7	4.44	101	10.1
Armepavine	LLOQ	90.1	14.5	95.0	6.71	104	5.32	88.6	11.6
	1	99.0	12.1	103	4.04	106	11.6	99.1	13.0
	20	118	3.44	114	8.58	98.2	8.28	91.8	8.79
	100	85.5	3.87	91.3	8.99	103	1.93	102	7.85
Pronuciferine	LLOQ	91.8	13.0	97.2	9.77	96.6	5.66	101	9.05
	2	95.6	9.36	93.9	3.91	100	5.32	103	4.39
	100	95.5	8.69	93.6	1.77	89.7	5.04	107	7.88
	500	86.9	4.62	86.8	1.00	85.7	3.00	110	10.3
Palmatine chloride (IS)	10	–	–	–	–	97.2	3.50	93.3	3.68

RSD, relative standard deviation.

#### Stability

The results for stability are shown in **Table 4**. It was indicated that the five alkaloids in rat plasma were stable under different storage conditions. As shown in **Table 4**, the five alkaloids were also stable in working solutions. In terms of the stock solution of IS, the remain was 114% with an RSD of 5.04%. Moreover, the range of remain was 101%–110% with RSDs less than 10% for stock solution of analytes.

**Table 4:** Stability of the five compounds (*n* = 6).

Compounds	Concentration (ng/ml)	Working solution		Autosampler for 24 h		Room temperature for 24 h		Freeze thaw cycles		−80°C for 1 month	
		Remain (%)	RSD (%)	Remain (%)	RSD (%)	Remain (%)	RSD (%)	Remain (%)	RSD (%)	Remain (%)	RSD (%)
Nunciferine	LLOQ	101	10.6	87.5	11.0	94.4	11.1	95.9	12.9	99.4	13.1
	2	92.5	5.47	89.7	4.50	96.5	6.42	92.5	4.07	96.3	4.48
	100	98.7	7.3	98.4	10.7	109	12.1	92.0	3.46	100	3.26
	500	108	8.14	85.4	3.27	89.3	7.67	85.3	4.24	91.4	2.61
O-nornuciferin	LLOQ	90.6	11.1	97.8	7.32	88.5	10.2	108	12.1	98.1	9.87
	1	94.6	10.3	104	9.71	101	11.6	106	13.8	107	11.8
	40	94.2	6.05	113	5.63	104	10.1	111	11.9	110	8.50
	200	109	8.78	97.8	6.47	102	13.6	96.4	6.41	107	6.22
Liriodenine	LLOQ	105	6.50	95.7	9.03	92.9	8.29	91.7	7.62	98.9	9.42
	1	96.9	5.91	86.0	6.82	87.9	12.2	86.9	7.88	107	9.18
	40	91.7	4.28	105	12.7	96.9	10.3	92.7	5.62	105	5.94
	200	112	6.11	96.8	6.11	102	6.75	90.5	5.59	104	6.55
Armepavine	LLOQ	96.3	7.54	87.6	8.88	91.5	7.02	103	8.40	109	8.58
	1	98.8	8.44	90.8	9.91	104	13.2	107	9.93	107	8.53
	20	95.8	5.22	114	13.2	118	10.8	111	7.73	113	5.24
	100	101	7.61	87.4	4.96	110	7.72	85.2	3.84	107	9.88
Pronuciferine	LLOQ	99.3	10.0	85.5	5.81	89.2	7.72	81.9	10.0	99.2	4.64
	2	92.9	7.33	88.3	4.23	106	12.9	93.3	6.30	99.0	7.54
	100	99.9	7.35	92.6	6.28	98.9	11.0	85.5	3.47	93.9	5.54
	500	105	7.36	87.5	5.32	87.1	3.18	87.2	3.03	90.4	3.81

#### Recoveries and Matrix Effects

The recoveries of the five alkaloids were in the range of 85.7% to 106% with RSDs less than 11.6% at four concentration levels. Moreover, the matrix effects of the five alkaloids ranged from 87% to 112% with RSDs less than 13%. The recovery and matrix effects of IS were 97.2% and 93.3%, respectively. It was indicated that the ionization of all compounds was not affected by interfering substances and the established method of plasma sample preparation with micro-SPE was efficient. These results are shown in **Table 3**.

#### Carryover

In terms of carryover effect, any peaks were not detected in three blank samples, which injected into HPLC-MS/MS just after the detection of ULOQ sample. Thus, the result indicated that carryover effects have no effect on the evaluation of the five alkaloids.

### Pharmacokinetic Application

**Figure 6:**
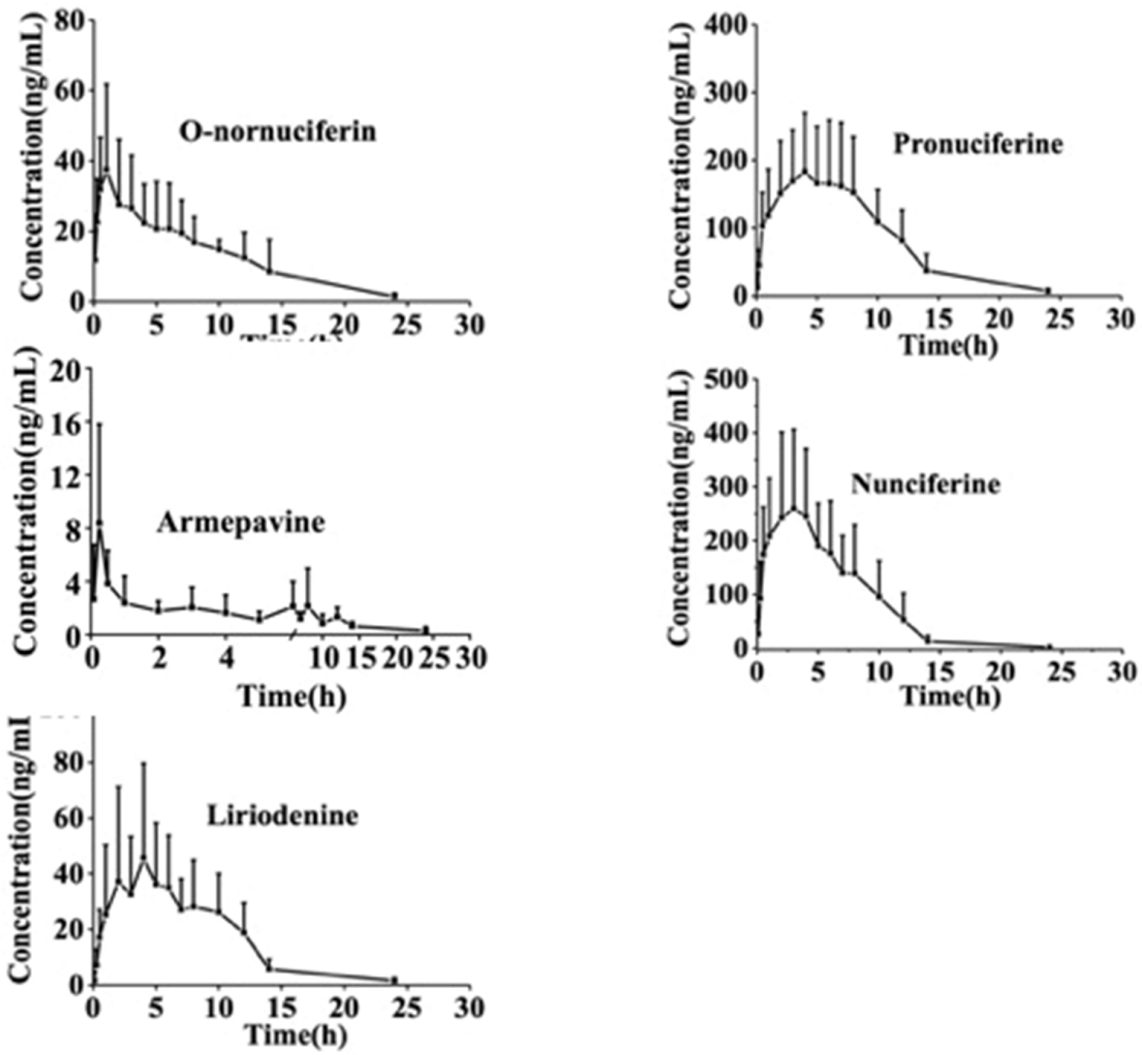
The mean plasma concentration-time profiles of five alkaloids after oral administration of *lotus leaf* extract (*n* = 10, mean ± SD).

The established method was used for the pharmacokinetics of the five orally administrated alkaloids (nunciferine, O-nornuciferin, liriodenine, armepavine, and pronuciferine) of *lotus leaf* extract at a dose of 2.4 g·kg^−1^ to rats. The pharmacokinetic outlines of the five alkaloids were represented by a one-compartmental model. The mean plasma concentration–time outlines are illustrated in **Figure 6**. As shown in **Table 5**, the C_max_ of armepavine was 8.78 ± 7.78 ng·ml^−1^ and the T_max_ was 0.30 ± 0.11 h. It was indicated that there was a fast absorption for armepavine after orally administrating the *lotus leaf* extract. Moreover, the T_max_ values of liriodenine and pronuciferine were 5.70 ± 2.83 and 4.90 ± 1.37 h, respectively. These results indicated that there was a slow absorption for liriodenine and pronuciferine.

**Table 5:** The main pharmacokinetic parameters of five compounds (*n* = 10).

Compounds	Nunciferine	O-nornuciferin	Liriodenine	Armepavine	Pronuciferine
T_max_ (h)	3.50 ± 1.84	2.60 ± 2.49	5.70 ± 2.83	0.30 ± 0.11	4.90 ± 1.37
C_max_ (ng/ml)	317.58 ± 175.05	42.08 ± 23.33	54.69 ± 31.84	8.78 ± 7.78	210 ± 87
T_1/2_ (h)	6.18 ± 3.10	6.67 ± 2.88	3.77 ± 1.15	5.22 ± 5.09	4.44 ± 1.88
AUC_(0–tn)_ (ng/ml*h)	2069 ± 919	319.8 ± 110.8	415.1 ± 199.8	28.60 ± 16.51	2031 ± 717
AUC_(0–∞)_ (ng/ml*h)	2142 ± 961	335.6 ± 107.3	429.0 ± 198.6	53.79 ± 34.65	2096 ± 704
MRT_(0–tn)_ (h)	5.49 ± 0.96	7.18 ± 1.88	7.11 ± 1.21	7.23 ± 1.93	7.43 ± 1.18
MRT_(0–∞)_ (h)	6.14 ± 1.47	8.47 ± 2.45	8.17 ± 1.86	31.94 ± 25.95	8.36 ± 1.98

On the other hand, the C_max_ was 317.6 ± 175.1 ng·ml^−1^ for nunciferine. It was demonstrated that the C_max_ of nunciferine was highest and the C_max_ of armepavine was lowest in five alkaloids. Moreover, AUCs_(0–24 h)_ of nunciferine and pronuciferine was 2069 ± 919 and 2031 ± 716 ng·ml^−1^, respectively. It was indicated that they possessed abundant plasma exposure.

### Comparison With Reference Methods

A few studies on one or two alkaloid of lotus leaf in pharmacokinetic study have been reported (Gu et al., 2014; Xu et al., 2015; Geng et al., 2018; Ye et al., 2018). Comparisons of the present work with other reported method are listed in **Table 6** in detail: First, compared with consumption of organic solvent in this work, there were less organic solvent in these reports (Xu et al., 2015; Geng et al., 2018). However, LLOQs of the present method were lower than those of reported methods. Second, consumptions of organic solvent in this work were more than these reports (Gu et al., 2014; Gu et al., 2014). However, matrix effects of the present method were more satisfactory than those of reported methods. Finally, the number of analytes in this work was more than those of analytes in these reported studies. Three of the measured five alkaloids components of *lotus leaf*, including O-nornuciferin, liriodenine, and pronuciferine, were determined by HPLC-MS/MS in this work for the first time. From the pharmacokinetic characteristics, compared to these reported studies (Gu et al., 2014; Xu et al., 2015; Ye et al., 2018), the T_max_ and T_1/2_ of nunciferine (3.50 ± 1.84 and 6.18 ± 3.10 h) was longer in this study. The AUCs_0–24__h_ of nunciferine have increased in this study. The extract of *lotus leaf* containing multitudinous compounds was orally administrated in this work. However, the alkaloid compound (nunciferine) or alkaloid fraction was orally administrated in these reports. Complex chemical composition in the extract has influenced the metabolism regularity of nunciferine. Therefore, the pharmacokinetic characteristics of nunciferine in this wok are not consistent with those in the aforementioned reports.

**Table 6:** Comparison with other methods for pharmacokinetic study of lotus leaf.

Analyte	Method	Type and volume of organic solvent in sample preparation	LLOQ (ng·ml^−1^)	Matrix effect	Reference
Nunciferine	HPLC-MS/MS	Ethyl acetate (1 ml), acetonitrile (100 µl)	0.1	98.3%–111.3%	Gu et al., 2014
Armepavine	UPLC-MS/MS	Acetonitrile (100 µl)	1	109.5%–113.7%	Geng et al., 2018
Nunciferine, N-nornuciferin	UPLC	Ethyl acetate (1.5 ml), 80% methanol (150 µl)	2.5	–	Ye et al., 2018
Nunciferine	UPLC-MS/MS	Acetonitrile–methanol (9:1, v/v, 150 µl)	2	88.5%–96.6%	Xu et al., 2015
Nunciferine, O-nornuciferin, liriodenine, armepavine, pronuciferine	Micro-SPE-HPLC-MS/MS	20% methanol (200 µl), acetonitrile (400 µl)	1, 0.5, 0.5, 0.5, 1	88.6%–112%	This work

In addition, the sample preparation with micro-SPE was firstly used to research the pharmacokinetic of *lotus leaf* extract. It was demonstrated that the established method was efficient and simple for simultaneously measuring multiple alkaloids in the pharmacokinetic study of *lotus leaf*.

## Conclusion

An efficient, novel, and sensitive method combined with micro-SPE was established for simultaneous determination of nunciferine, O-nornuciferin, liriodenine, armepavine, and pronuciferine after oral administration of *lotus leaf* extract to rats. This method provided satisfactory recoveries and matrix effects for all analytes. In addition, the optimized sample preparation method has a few advantages, including lower organic solvent consumption, easier operation, and being more efficient. Moreover, the pharmacokinetic data indicated that the absorption and elimination of armepavine was fast. Nunciferine and pronuciferine possessed abundant plasma exposure. In summary, the established method was successfully used to investigate pharmacokinetics of the five orally administrated alkaloids of *lotus leaf* extract to rats, which could help us to better understand the pharmacokinetics and evaluate the clinical efficiency of *lotus leaf*.

## Data Availability Statement

All datasets generated for this study are included in the manuscript/supplementary files.

## Ethics Statement

The animal study was reviewed and approved by The Animal Ethics Committee of Tianjin University of Traditional Chinese Medicine.

## Author Contributions

Conceptualization: Y-XC. Data curation: SZ, YG, JL, and Y-XC. Formal analysis: SZ, YG, XC, JL, HW, and XY. Funding acquisition: Y-XC. Investigation: SZ and YG. Writing—original draft: SZ, JL, Y-XC. Writing—review and editing: XG and Y-XC.

## Conflict of Interest

The authors declare that the research was conducted in the absence of any commercial or financial relationships that could be construed as a potential conflict of interest.
